# Perfluorooctanoic acid exposure for 28 days affects glucose homeostasis and induces insulin hypersensitivity in mice

**DOI:** 10.1038/srep11029

**Published:** 2015-06-12

**Authors:** Shengmin Yan, Hongxia Zhang, Fei Zheng, Nan Sheng, Xuejiang Guo, Jiayin Dai

**Affiliations:** 1 Key Laboratory of Animal Ecology and Conservation Biology, Institute of Zoology, Chinese Academy of Sciences, Beijing 100101, P.R. China; 2 Shanxi Key Laboratory of Ecological Animal Science and Environmental Medicine, Shanxi Agricultural University, Taigu 030801, P.R. China; 3 State Key Laboratory of Reproductive Medicine, Nanjing Medical University, Nanjing 210029, P.R. China

## Abstract

Perfluoroalkyl acids (PFAAs) are widely used in many applications due to their unique physical and chemical characteristics. Because of the increasing prevalence of metabolic syndromes, including obesity, dyslipidemia and insulin resistance, concern has arisen about the roles of environmental pollutants in such diseases. Earlier epidemiologic studies showed a potential association between perfluorooctanoic acid (PFOA) and glucose metabolism, but how PFOA influences glucose homeostasis is still unknown. Here, we report on the modulation of the phosphatidylinositol 3-kinase-serine/threonine protein kinase (PI3K-AKT) signaling pathway in the livers of mice after 28 d of exposure to PFOA. Compared with normal mice, PFOA exposure significantly decreased the expression of the phosphatase and tensin homologue (PTEN) protein and affected the PI3K-AKT signaling pathway in the liver. Tolerance tests further indicated that PFOA exposure induced higher insulin sensitivity and glucose tolerance in mice. Biochemical analysis revealed that PFOA exposure reduced hepatic glycogen synthesis, which might be attributed to gluconeogenesis inhibition. The levels of several circulating proteins were altered after PFOA exposure, including proteins potentially related to diabetes and liver disease. Our results suggest that PFOA affected glucose metabolism and induced insulin hypersensitivity in mice.

Perfluoroalkyl acids (PFAAs) are a family of anthropogenic compounds used worldwide in a number of applications due to their unique physical and chemical characteristics^1^. Perfluorooctanoic acid (PFOA) is one of the most widely known PFAAs found in human and wildlife serum, with earlier toxicological studies showing its evident effects on body weight, liver weight, serum cholesterol and mortality in rodents^1^.

Peroxisome proliferator activated receptors (PPARs) mediate the effects of eicosanoids, fatty acids and synthetic peroxisome proliferators on gene transcription and play specific roles in lipid metabolism and inflammation regulation^2^,^3^,^4^. Three PPAR isotypes, PPARα, β/δ and γ, have been identified, with PPARα highly expressed in the liver^4^. PFOA has been reported to induce liver enlargement along with alteration of lipid metabolism genes in rodents. Due to its significant effect on fatty acids β-oxidation, ketogenesis and systemic lipid metabolism, PPARα activation is the most likely reason that PFOA induces hepatomegaly and hepatic lipid metabolism^1^,^3^,^5^. Medical surveillance research and experimental studies have investigated the potential effects of PFOA on liver and lipid metabolism. PFOA exposure appears to be positively associated with serum alanine aminotransferase (ALT) levels and negatively associated with serum high-density lipoprotein cholesterol (HDL-C) levels in fluorochemical production workers^6^,^7^. Triglycerides are also positively associated with PFOA in humans, though not consistently across locations^8^,^9^.

Different from lipid metabolism, only a small number of studies on the effects of PFAAs on glucose metabolism have been reported. Due to the rising prevalence of metabolic syndromes, including obesity, dyslipidemia and insulin resistance, there is growing interest in whether environmental pollutants are involved in such diseases^10^. Medical surveillance studies have implied that environmental exposure might be associated with diabetes mellitus, a metabolic disease characterized by high blood glucose levels^11^,^12^. However, several epidemiological studies have reported that PFOA and other PFAAs are not associated with diabetes, but might influence glucose metabolism in humans^10^,^11^,^13^,^14^,^15^. Clearly, the association between PFAAs and diabetes remains uncertain, and the study on mechanisms of the potential effects of PFOA on the glucose homeostasis is necessary.

Phosphatidylinositol 3-kinases (PI3Ks) can be divided into three classes based on their structural and biochemical features. Class IA PI3Ks (including p110α, p110β and p110δ), as well as the p85 regulatory subunits (including p85α, p85β, p55γ, p55α and p50α), have been suggested to play key roles in the metabolic functions of insulin^16^,^17^,^18^. Phosphoinositide-dependent kinase 1 (PDK1) is one of the best characterized signaling molecules regulated by PI3K and it consequently activates serine/threonine protein kinase (AKT, also known as protein kinase B, PKB), which is important in the transmission of the insulin signal^16^,^19^. Because of the key role the liver plays in glucose homeostasis and the evident toxicological effects of PFOA on the liver, we hypothesized that PFOA could disturb glucose homeostasis through interference with hepatic glucose metabolism. Accordingly, we assessed the modulation of the PI3K-AKT signaling pathway in mouse liver after 28 d of PFOA exposure, and further investigated the effects of PFOA on glucose metabolism through tolerance tests and serum proteomics. We found that PFOA exposure for 28 d affected glucose metabolism and increased insulin sensitivity in mice.

## Results

### PFOA exposure increased phosphorylation of AKT along with decreased expression of PTEN protein in mouse liver

**Figure 1:**
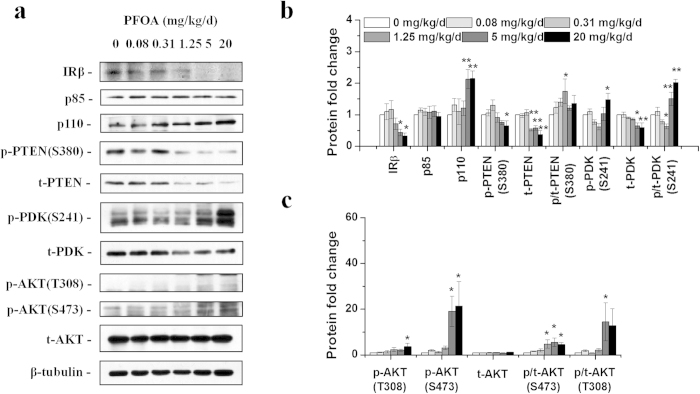
PFOA exposure decreased the protein expression of PTEN and modulated the PI3K-AKT signaling pathway. (**a**) Representative western blotting images of liver lysates prepared from mice exposed to different doses of PFOA using antibodies against IRβ, p85, p110, phospho-PTEN (S380), total PTEN, phospho-PDK1 (S241), total PDK1, phospho-AKT (T380), phospho-AKT (S473) and total AKT protein. In figures reported here, p- indicates phospho- and t- indicates total, as mentioned above. (**b**) and (**c**) Relative fold change of band densities (n = 3). Data are means ± SE. Significantly different from control group (*p < 0.05, **p < 0.01).

**Figure 2:**
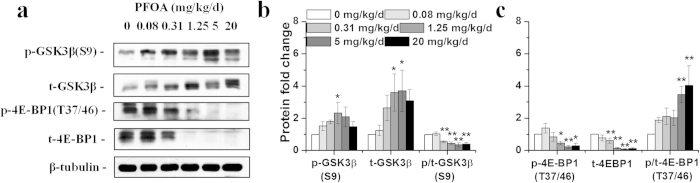
PFOA exposure altered phosphorylation of AKT substrates. (**a**) Western blot of liver lysates prepared from mice exposed to different doses of PFOA with antibodies of phospho-GSK3β (S9), total GSK3β, phospho-4E-BP1 (T37/46) and total 4E-BP1. (**b**) and (**c**) Relative fold change of band densities (n = 3). Data are means ± SE. Significantly different from control group (*p < 0.05, **p < 0.01).

In our previous study, we observed significant liver enlargement and lipid metabolism dysfunction in mice exposed to PFOA for 28 d^20^,^21^. To investigate the effects of PFOA on hepatic glucose metabolism, key factors of the PI3K-AKT signaling pathway in livers were analyzed using western blotting. A significant reduction in insulin receptor β (IRβ), the transmembrane IR subunit with tyrosine kinase activity^22^, was observed in mouse livers after exposure to PFOA at doses of 5 and 20 mg/kg/d (Fig. 1a,b). The protein expression of p85, a regulatory subunit of Class IA PI3K^18^, was not significantly changed after PFOA exposure; however, the expression of catalytic subunit p110 was significantly increased in livers of mice exposed to PFOA at doses of 5 and 20 mg/kg/d (Fig. 1a,b). Interestingly, the protein expressions of the phosphatase and tensin homologue (PTEN), a PI3K-AKT signaling pathway repressor, were significantly deceased in livers of mice treated with 1.25 mg/kg/d and higher doses of PFOA (Fig. 1a,b). PDK1 exhibited greater phosphorylation at site Ser 241, and its total protein was decreased (Fig. 1a,b). Proteins phosphorylated at two major AKT phosphorylated sites (Thr 308 and Ser 473) increased after PFOA exposure, especially at high doses when even IRβ decreased (Fig. 1a,c). The phosphorylation level of glycogen synthase kinase 3-beta (GSK3β), a direct target of AKT, showed a humble increase; however, total GSK3β protein increased significantly (Fig. 2a,b). Since AKT can activate the mammalian target of rapamycin (mTOR) complex 1, a familiar mTORC1 substrate, that is, eukaryotic translation initiation factor 4E-binding protein 1 (4E-BP1), was also detected. Both phosphorylated and total proteins of 4E-BP1 were less expressed after PFOA exposure, with the ratio of phosphorylated 4E-BP1 significantly increased at high doses of PFOA (Fig. 2a,c). Another important pathway in hepatic glucose metabolism, the 5'-AMP-activated protein kinase (AMPK) pathway, was also analyzed; however, the expressions of phosphorylated and total AMPK proteins exhibited little change after PFOA exposure (Fig. S1). The mRNA level of glucose-6-phosphatase catalytic subunit (G6pc) decreased in livers of mice dosed with 0.31 mg/kg/d PFOA for 28 d and gradually increased in groups treated with higher doses (Fig. S2).

### PFOA exposure induced insulin hypersensitivity in mice

**Figure 3:**
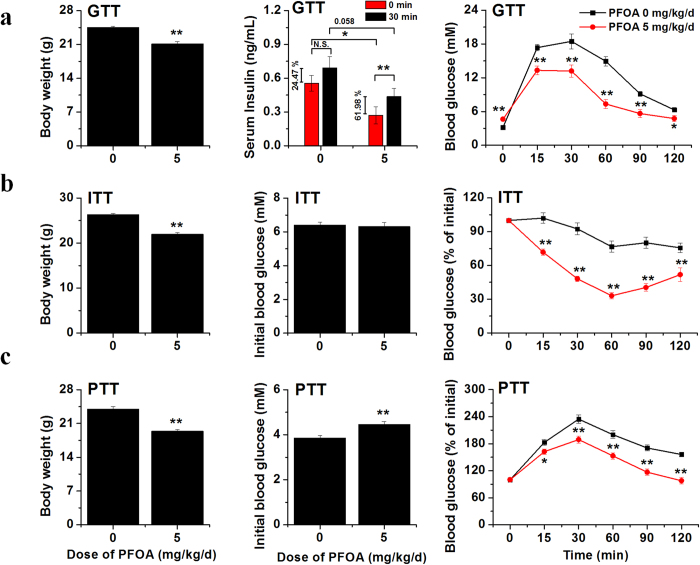
Tolerance tests of mice after PFOA exposure. (**a**) Glucose tolerance tests of mice exposed to PFOA at 5 mg/kg/d (n = 10). Mice were starved for 16 h and weighed (left). Serum insulin concentrations before and 30 min after glucose injection were analyzed (middle) and blood glucose concentrations were measured before and after glucose injection (right). (**b**) Insulin tolerance tests of mice exposed to PFOA at 5 mg/kg/d (n = 10). Mice were starved for 4 h and weighed (left). Glucose concentrations before insulin injection were measured (middle), with blood glucose concentration after insulin injection determined as a percentage of the concentration before injection (right). (**c**) Pyruvate tolerance tests of mice exposed to PFOA at 5 mg/kg/d (n = 10). Mice were starved for 16 h and weighed (left). Glucose concentration before pyruvate injection was measured (middle), with blood glucose concentration after pyruvate injection determined as a percentage of the concentration before injection (right). Data are means ± SE. Significantly different from control group (*p < 0.05, **p < 0.01).

**Figure 4:**
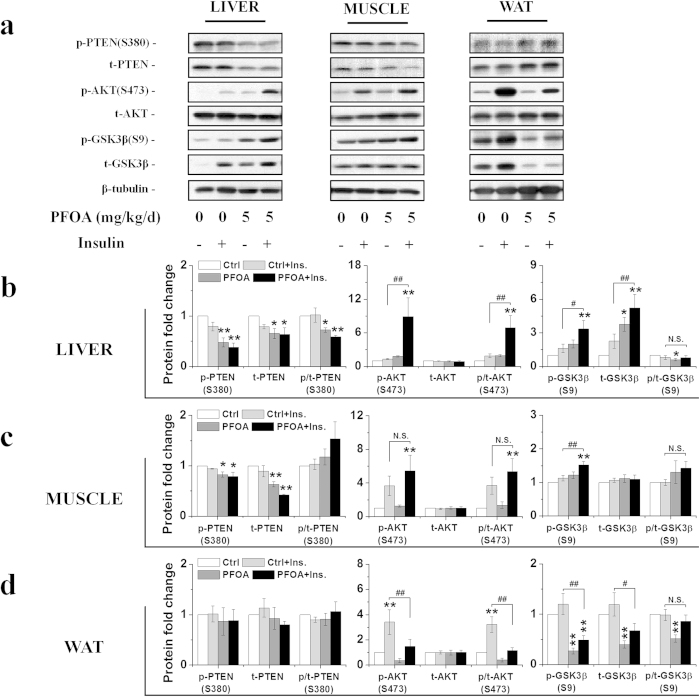
PFOA exposure increased insulin sensitivity in the livers and muscles of mice. (**a**) Western blots of liver, muscle and white adipose tissues (WATs) lysates prepared from PFOA-exposed mice treated with insulin with antibodies of phosphorylated and total PTEN, AKT and GSK3β. (**b**) Relative fold change of band densities from livers (n = 3). (**c**) Relative fold change of band densities from muscles (n = 3). (**d**) Relative fold change of band densities from WATs (n = 3). Data are means ± SE. Significantly different from control group (*p < 0.05, **p < 0.01, N.S., not significant).

Based on the effects of PFOA on the PI3K-AKT signaling pathway, we hypothesized that PFOA exposure might modify insulin sensitivity in mice. Tolerance tests were performed to further analyze the effects of PFOA on glucose homeostasis. Results from glucose tolerance tests (GTTs) showed that mice exposed to 5 mg/kg/d PFOA exhibited lower serum insulin, higher basal blood glucose, as well as higher glucose tolerance, compared with those of the control group after glucose injection (Fig. 3a). The blood glucose levels were close between the control and PFOA-treated mice before the insulin tolerance tests (ITTs). After insulin injection, unlike the slow reduction in the control mice, blood glucose concentration in the PFOA-exposed mice reduced rapidly, which implied an underlying insulin hypersensitivity in PFOA-exposed mice. Pyruvate tolerance tests (PTTs) were also performed to analyze the potential effects of PFOA on gluconeogenesis. The blood glucose concentration was higher in PFOA-exposed mice, with a slower and lower blood glucose increase observed after pyruvate injection (Fig. 3c). Two days after the tolerance tests, the mice were sacrificed and organs were weighed. Results showed that livers weights were increased after PFOA exposure and both white and brown adipose tissue weights were significantly decreased (Table S2). Serum biochemical levels were also analyzed and levels of all biochemical factors were altered in a similar pattern, as per our previous research^20^ (Table S2). To find molecular evidence directly supporting PFOA increasing insulin sensitivity, mice exposed to PFOA for 28 d were stimulated with insulin for 10 min after 4 h starvation, and livers, skeletal muscles and gonadal white adipose tissues (WATs) were collected to analyze the stimulation of the PI3K-AKT signaling pathway. As expected, increased phosphorylation of AKT and GSK3β after insulin stimulation was observed in the livers of mice exposed to PFOA compared with those of the control group (Fig. 4a,b). Surprisingly, muscle, another important organ in energy metabolism, was also more sensitive to insulin and had lower PTEN protein levels after PFOA exposure compared with that of the control group (Fig. 4a,c). The PI3K-AKT signaling pathway was also modulated in WATs after PFOA exposure; however, unlike liver and muscle, the pathway seemed depressed and less sensitive to insulin after PFOA exposure (Fig. 4a,d).

### PFOA exposure altered carbohydrate metabolism

**Figure 5:**
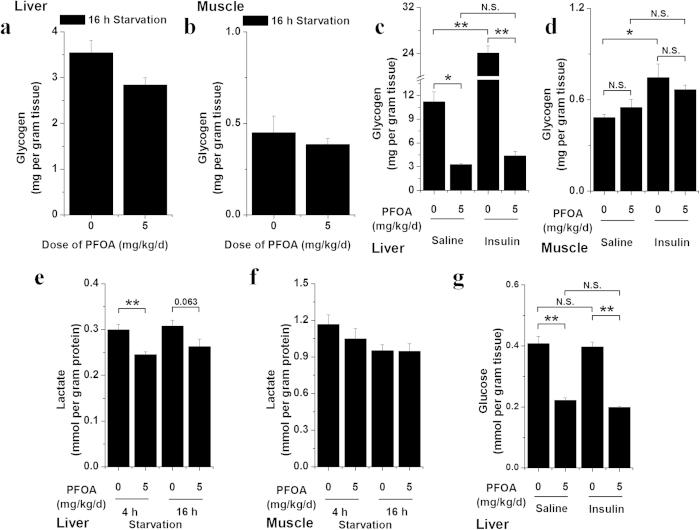
PFOA exposure disturbed glycogen metabolism in mice. (**a**) Glycogen content in livers of mice dosed with 5 mg/kg/d PFOA after 16 h starvation (n = 5). (**b**) Glycogen content in muscles of mice dosed with 5 mg/kg/d PFOA after 16 h starvation (n = 5). (**c**) Glycogen content in livers of mice dosed with 5 mg/kg/d PFOA and treated with saline or insulin after 4 h starvation (n = 5). (**d**) Glycogen content in muscles of mice dosed with 5 mg/kg/d PFOA and treated with saline or insulin after 4 h starvation (n = 5). (**e**) Lactic acid content in livers of mice dosed with 5 mg/kg/d PFOA after 4 or 16 h starvation (n = 5). (**f**) Lactic acid content in muscles of mice dosed with 5 mg/kg/d PFOA after 4 or 16 h starvation (n = 5). Data are means ± SE. Significantly different from control group (*p < 0.05, **p < 0.01, N.S., not significant).

The liver plays a central role in blood glucose level maintenance by balancing glucose uptake and storage via glycogenesis and glucose release through glycogenolysis and gluconeogenesis^23^. Due to the significant effects of PFOA on increasing insulin sensitivity, we further analyzed the hepatic and muscular carbohydrate stores in mice after 28 d of exposure to PFOA. Biochemical analysis revealed a modest and nonsignificant reduction in glycogen content in the livers and muscles of PFOA-exposed mice after 16 h starvation (Fig. 5a,b). Livers from PFOA-exposed mice after 4 h starvation showed a marked reduction in glycogen content and significantly lower glycogen increase after insulin stimulation compared with that of the control group (Fig. 5c). There was no significant difference in muscular glycogen content between the control and PFOA-treated mice after 4 h starvation, and this was irrelevant to insulin stimulation. However, glycogen content was significantly increased after insulin stimulation in the control mice, but not in the PFOA-exposed mice (Fig. 5d). The lower lactate content in livers from PFOA-exposed mice and no marked lactate change in muscles (Fig. 5e,f) indirectly suggested that glycolysis was comparable in both groups of animals. Hepatic glucose content was also analyzed in insulin-stimulated mice and significantly reduced in mice after PFOA exposure (Fig. 5g). These results suggest a potential impairment in the carbohydrate metabolism of mice after PFOA exposure.

### PFOA exposure changed serum protein profiles

**Figure 6:**
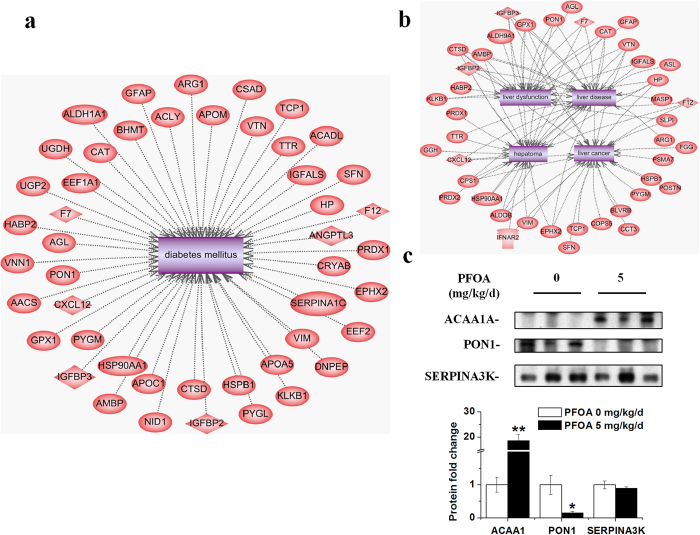
PFOA exposure altered serum protein profiles. (**a**) Network of proteins related to diabetes differentially expressed in serum of mice exposed to PFOA. (**b**) Network of proteins related to liver functions or diseases differentially expressed in the serum of mice exposed to PFOA. (**c**) Western blot of serum from PFOA-exposed mice validated the iTRAQ results for ACAA1A, PON1 and SERPINA3K. Bar graph for relative fold change of band densities is presented below protein bands. Data are means ± SE. Significantly different from control group (*p < 0.05, **p < 0.01).

Many organs are involved in glucose homeostasis maintenance, and the effects of PFOA on glucose metabolism may be reflected in circulation due to its contact with blood. To further understand the effects of PFOA on glucose homeostasis, we analyzed the profiles of serum proteins using isobaric tags for relative and absolute quantitation (iTRAQ) in mice exposed to PFOA. iTRAQ analysis was performed on four individual serum samples from the control and PFOA-exposed mice, respectively, and 172 differentially expressed proteins (133 up-regulated and 39 down-regulated) were identified (Table S3). The differentially expressed proteins were further analyzed for interaction with the most familiar glucose metabolism dysfunction disease, diabetes mellitus, using Pathway Studio software via the ResNet database. Forty-seven proteins were found to be related to diabetes mellitus (Fig. 6a and Table 1). Remarkably, insulin-like growth factor (IGF)-binding protein (IGFBP) 2 and 3, which are important in regulating metabolic homeostasis^24^,^25^,^26^,^27^, showed 1.86- and 1.57-fold increases, respectively, in the serum of mice exposed to PFOA (Fig. 6a and Table 1). Because of the representative effects of PFOA on the liver, we also searched the interaction of differentially expressed serum proteins with liver function, and found 23 proteins related to hepatoma, 24 related to liver cancer, 20 related to liver disease and 18 related to liver dysfunction (Fig. 6b and Table 1). Three proteins were selected to validate the iTRAQ results via western blotting, and another three samples from each group, which were not used for iTRAQ, were used to validate the results. Among the three proteins, the levels of ACAA1A increased and PON1 decreased, as also shown in the iTRAQ results, in the serum of mice after PFOA exposure at 5 mg/kg/d (Fig. 6c). The level of SERPINA3K decreased in the iTRAQ results but no significant density fold change was found when analyzed with western blotting (Fig. 6c). Interestingly, the levels of the three proteins were also altered in mice exposed to PFOA at 1.25 mg/kg/d, and PON1 and SERPINA3K showed a decrease in mice dosed with 0.31 mg/kg/d PFOA (Figs. S3a and b).

## Discussion

There is growing interest in the role of environmental pollutants in the prevalence of metabolic syndromes^10^,^12^,^28^. Environmental exposure to persistent organic pollutants (POPs), arsenic, bisphenol A, phthalates, organotins and non-persistent pesticides is reportedly associated with diabetes^12^. Medical surveillance studies have also implied a potential connection between PFAAs and diabetes or glucose homeostasis^10^,^11^,^13^,^14^,^15^. However, the molecular mechanism of the PFAA effects on glucose metabolism remains unclear. The PI3K-AKT signaling pathway is highly conserved and plays a pivotal role in the metabolic actions of insulin^16^,^19^. We analyzed the modulation of the PI3K-AKT signaling pathway in the livers of mice exposed to PFOA for 28 d, and found that it was altered, especially at doses higher than 1.25 mg/kg/d, with AKT activation and even the IRβ levels decreased. PFOA content in serum and livers was analyzed and documented in our previous reports^20^,^21^, which demonstrated its accumulation in mice in a dose dependent manner after exposure. Our earlier study showed a median level of 1.64 μg/mL PFOA in the blood of occupational workers^7^, which is similar to the serum level of PFOA in mice from the 0.08 mg/kg/d group (2.24 μg/mL)^20^. Taking the serum PFOA content and our present results together, the effects of PFOA on the PI3K-AKT signaling pathway appeared at exposure doses higher than realistic exposure in occupational workers. Nonetheless, we cannot exclude whether the effects of PFOA on glucose metabolism may appear after long-term, low dose PFOA exposure in mice as well as in humans.

In accordance with the alteration of the PI3K-AKT signaling pathway after PFOA exposure, mice were more sensitive to insulin and more tolerant to glucose. Insulin-stimulated mice further demonstrated AKT activation and insulin hypersensitivity in both livers and muscles after PFOA exposure. In the glucose tolerance tests, although the basal serum insulin was lower in PFOA-exposed mice, serum insulin increased after glucose injection in both groups, and compared with the control mice (24.47%) PFOA-exposed mice induced more serum insulin after glucose treatment (61.38%). These results indirectly suggest the function of islets was not impaired by PFOA and lower serum insulin in PFOA-exposed mice might be an adaptation to high insulin sensitivity. Similar to the results from earlier research^29^, we also observed that both white and brown adipose tissue weights were significantly decreased following exposure, which may result from the activation of PPARα in the liver by PFOA. Different from liver and muscle, we found no significant change in PTEN levels but a significant reduction of phosphorylated AKT as well as both phosphorylated and total GSK3β. According to previous studies, the structure of adipose tissues and size of adipocytes are associated with adipose tissue functions^30^,^31^, and whether the atrophy of WAT led to the reduction in AKT phosphorylation and GSK3β still needs to be further explored.

The PI3K-AKT signaling pathway is tightly controlled by a multistep process, in which PTEN, a lipid and protein phosphatase, plays an important role^17^,^19^. PTEN negatively regulates the PI3K-AKT signaling pathway via its principal catalytic function to dephosphorylate phosphatidylinositol3,4,5trisphosphate (PIP3), a messenger that contributes to partial AKT activation^17^,^19^. It is interesting to note that the loss of PTEN protein was found in the livers and muscles of mice, especially in the higher PFOA exposure groups. Earlier studies suggest that the loss of PTEN in the liver could impair glucose homeostasis and result in fatty liver or even hepatocellular carcinomas, with genes involved in lipid metabolism and the PI3K-AKT signaling pathway altered in liver specific PTEN-deficient mice^32^,^33^. Effects of PFOA on lipid metabolism have been studied previously^34^, and several genes involved in lipid metabolism were also found to be altered after PFOA exposure in our earlier work^21^. Other than the effects on metabolism, AKT also regulates cell growth and survival^35^. Upon PTEN loss, a potent derepression of the PI3K-AKT signaling pathway may stimulate cell proliferation^17^. The important roles of PTEN in tumor suppression is supported by the study of *PTEN*-deficient or -mutated mouse models, and in several sporadic and heritable tumor types PTEN has been identified as lost or mutated^36^. Taking into account the tumorigenicity of PFOA in rodents^1^,^34^, whether PTEN loss in mice plays an important role in PFOA toxicological effects is worth further study. Insulin resistance is one significant characteristic of type 2 diabetes^37^, and it appears that PFOA-induced insulin hypersensitivity in the liver and muscle could be of benefit to the defense of type 2 diabetes. However, PTEN loss in the liver and muscle of mice exposed to PFOA might be the pivotal reason for high insulin sensitivity. Considering the long-term effects of PTEN loss^32^,^33^, further investigation is needed to clarify whether insulin hypersensitivity is just one phenomenon in conjunction with tumor development induced by PFOA exposure in rodents.

Under hormonal control, hepatocytes produce glucose through stimulating gluconeogenesis and glycogenolysis in the fasting state; however, hepatocytes store glucose by suppressing hepatic gluconeogenesis and glycogenolysis and increasing hepatic glycogen synthesis in the feeding condition^38^. These controls include transcriptional regulation of rate limiting enzymes and enzyme activity modulation^38^. In the present study, liver and muscle carbohydrates were analyzed by biochemical methods. Unlike previous reports with high hepatic glycogen content in liver specific PTEN-deficient mice, glycogen content in both the livers and muscles of mice exposed to PFOA exhibited no significant change after long starvation. Insulin stimulations also demonstrated a reduction in the hepatic glycogen content in mice exposed to PFOA and no significant glycogen synthesis was found in either the livers or muscles of PFOA-exposed mice. Insulin stimulates glucose uptake in muscles and fats, but promotes glycogen synthesis and glycolysis without stimulating glucose uptake in livers^16^,^33^. Hepatic glycogen deposition is an important process during blood glucose level maintenance, and is initiated by several stimuli, including increased hepatic glucose uptake, metabolites and hormones in the portal vein and increased glyconeogenesis^39^. The increased activation of AKT in the livers and muscles of mice exposed to PFOA after insulin stimulation compared with that of the control group, as well as the insulin tolerance test results, supported the increased insulin sensitivity observed in mice after PFOA exposure. In our previous study^21^, we found no significant changes in the hepatic mRNA levels of several enzymes involved in glucose metabolism, including aldolase B (Aldob), phosphoenolpyruvate carboxykinase 1 (Pepck) and pyruvate kinase in livers (L-pk) exposed to PFOA for 28 d. In the present study, although the G6pc mRNA levels were differentially expressed in the livers of mice dosed with 0.31 mg/kg/d and 20 mg/kg/d PFOA for 28 d, there was no significant change at the dose of 5 mg/kg/d, which was used for further study. These results suggest that the reduction in hepatic glycogen storage may not be caused by transcriptional regulation of these rate limiting enzymes. The lactate content in livers and muscles also indirectly suggested that glycolysis was comparable in both groups of animals. There was also no redundant glucose in the livers of mice after PFOA exposure due to repressed glycogen synthesis. Earlier reports demonstrated that gluconeogenesis inhibition could induce modest postprandial glycogen synthesis to take place^39^,^40^. Our pyruvate tolerance test results indirectly suggested that PFOA exposure impaired the gluconeogenesis process; however, whether gluconeogenesis inhibition contributes to a reduction in hepatic glycogen content still needs further study.

Glucose metabolism is predominantly regulated by serum hormones, including insulin and glucagon, and many other factors. The IGF system, which includes two IGF ligands, two IGF receptors, six binding proteins and several binding protein proteases, constitutes a complex molecular network and plays an important role in mammalian growth and development^25^,^41^. IGFs are involved in cell destinies and metabolic actions, and the IGFBP family is best known for regulating IGF activity^41^. Results from serum proteomic analysis showed IGFBP 2 and 3 levels were altered in mouse serum after PFOA exposure. Many studies have indicated that circulating IGFBP levels and the ratio of IGFs to IGFBPs play important roles in metabolic homeostasis and their fluctuations might correlate to metabolic syndromes and cancers^24^,^25^,^26^,^27^. However, whether the alteration of IGFBP 2 and 3 concentrations in PFOA-exposed mouse serum plays a direct role in PFOA toxicological effects, especially on glucose metabolism, still needs further exploration. Accumulating evidence indicates protein profiling of blood, serum and plasma varies between healthy individuals and those with insulin resistance or type 2 diabetes^42^. Earlier proteomic research on a type 2 diabetes mouse model showed serine protease inhibitor A3K (SERPINA3K) levels were elevated in the serum of mice compared with those in normal mice^43^. SERPINA3K is suggested to inhibit proteolytic activities of tissue kallikrein and potentially participate in the regulation of vasodilation and local blood flow^44^. In a diabetic rat model, SERPINA3K levels were decreased in the retinas, with this alteration possibly contributing to diabetic retinopathy^44^. We also found decreased circulating SERPINA3K levels in mice exposed to PFOA for 28 d, and its potential actions in PFOA-induced effects on glucose metabolism justifies further study. Besides the possible involvement of proteins in glucose metabolism, the levels of proteins correlated with liver disease also fluctuated in the serum of mice after PFOA exposure, further reflecting potential biomarkers for PFOA exposure in serum and that probable long distance regulation processes between organs may contribute to PFOA toxicological effects.

To our knowledge, this is the first systematic study to report on the effects of PFOA on glucose homeostasis. Our research indicated that PFOA exposure induced AKT activation along with loss of the PTEN protein in both the livers and muscles of mice. Further study showed PFOA increased insulin sensitivity and disturbed hepatic glycogen synthesis. Serum proteomic analysis demonstrated that several protein levels fluctuated after PFOA exposure, including proteins reported as potentially related to diabetes and liver disease. Results from this study implied that the effects of PFOA on glucose homeostasis were not confined to a single organ such as the liver, but may involve many organs that participate in glucose homeostasis maintenance. Some questions remain unresolved in our present research, however, including the exact molecular mechanisms of hepatic glycogen reduction after PFOA exposure and the functions of differentially expressed circulating proteins. Further investigations are needed to elucidate the potential correlation between PFOA or other PFAAs and glucose metabolism.

## Methods

### Chemicals and antibodies

Perfluorooctanoic acid (PFOA, CAS number 335-67-1, 96% purity) was obtained from Sigma-Aldrich (St. Louis, MO, USA). Anti-phospho-AKT (Ser473) rabbit monoclonal antibody, anti-phospho-AKT (Thr308) rabbit monoclonal antibody, anti-AKT rabbit monoclonal antibody, anti-phospho-PDK1 (Ser241) rabbit monoclonal antibody, anti-PDK1 rabbit polyclonal antibody, anti-phospho-PTEN (Ser308) rabbit polyclonal antibody, anti-PTEN rabbit monoclonal antibody, anti-PI3 Kinase p85 rabbit monoclonal antibody, anti-PI3 Kinase p110α rabbit monoclonal antibody, anti-phospho-GSK-3β (Ser9) rabbit monoclonal antibody, anti-phospho-4E-BP1 (Thr37/46) rabbit monoclonal antibody, and anti-4E-BP1 rabbit monoclonal antibody were purchased from Cell Signaling Technology (Beverly, MA, USA). Anti-GSK-3β rabbit polyclonal antibody was purchased from Signalway Antibody LLC (College Park, MD, USA). Anti-ACAA1 mouse monoclonal antibody and anti-PON1 mouse monoclonal antibody were purchased from Abcam (Cambridge, MA, USA). Anti-IRβ rabbit polyclonal antibody, anti-SERPINA3K goat polyclonal antibody, HRP-conjugated goat anti-mouse, HRP-conjugated goat anti-rabbit and HRP-conjugated donkey anti-goat secondary antibodies were purchased from Santa Cruz Biotechnology (Santa Cruz, CA, USA). Anti-β-tubulin mouse monoclonal antibody was purchased from Beijing CoWin Bioscience Co., Ltd. (Beijing, China). All other chemicals used were of the highest grade commercially available.

### Animal treatment

Male Balb/c mice (age 6–8 weeks) were purchased from Beijing Vital River Experimental Animals Centre (Beijing, China). Experimental manipulations are described in our previous study^20^. Briefly, mice were randomly divided into six groups and treated with PFOA diluted in Milli-Q water at doses of 0, 0.08, 0.31, 1.25, 5 and 20 mg/kg/d via gavage for 28 d in accordance with earlier studies and previous experiments. This study was conducted in accordance with the Animal Ethics Committee of the Institute of Zoology, Chinese Academy of Sciences. The institute does not issue a number or ID to any animal study, but the ethical committee guides the animal use and conduct.

### Glucose, pyruvate and insulin tolerance tests and insulin stimulations

For practical reasons, another 40 mice were randomly assigned into two groups and dosed with either Milli-Q water or 5 mg/kg/d PFOA. Ten mice from each group were used for glucose and insulin tolerance tests at day 25 and day 27, respectively, and sacrificed at day 29 as per our previous study^20^. Another 10 mice from each group were used for pyruvate tolerance tests at day 27 and insulin stimulations at day 29. According to earlier studies and our previous experiments, glucose tolerance tests (GTTs) were performed via intraperitoneal glucose injection at a dose of 2 g/kg after a 16-h fast, pyruvate tolerance tests (PTTs) were performed via intraperitoneal pyruvate injection at a dose of 1 g/kg after a 16-h fast, and insulin tolerance tests (ITTs) were performed via intraperitoneal insulin injection at a dose of 0.75 U/kg after a 4-h fast^45^. Initial blood glucose was measured with blood taken from the tail vain before GTTs, PTTs and ITTs using an Accu-Chek^®^ Performa Glucometer (Roche Diagnostics GmbH, Mannheim, Germany) according to the manufacturers’ recommendations, with blood glucose measured again 15, 30, 60, 90 and 120 min after injection. Blood samples from the retro-orbital sinus were carefully collected using a capillary tube before GTTs and 30 min after injection, and were clotted for 1 h at room temperature. Serum samples were collected to measure insulin concentration using a mouse ultrasensitive insulin ELISA kit (ALPCO diagnostics, NH, USA) according to the manufacturer’s recommendations. Insulin stimulations were performed via intraperitoneal insulin injection at a dose of 0.75 U/kg for 10 min after a 4-h fast and the mice were subsequently sacrificed by cervical dislocation. Tissues were removed and immediately frozen in liquid nitrogen after sacrifice. Serum and tissues were stored at −80 °C until further use. All animal treatment protocols were approved by the Committee on the Ethics of Animal Experiments from the Institute of Zoology, Chinese Academy of Sciences.

### Serum biochemical assay

Serum lipids and enzymes were detected using a HITAC7170A automatic analyzer (Hitachi, Japan) following standard spectrophotometric methods (n = 10).

### Glycogen, lactate and glucose content measurement

The glycogen, lactate and glucose content in mouse livers and muscles after PFOA exposure were analyzed using a glycogen assay kit, lactate assay kit and glucose assay kit (Nanjing Jiancheng Bioengineering Institute, Nanjing, China), respectively, according to the manufacturer’s instructions.

### Real-time PCR analysis

Mouse liver total RNAs (n = 6) were isolated using TRIzol (Life Technologies-Invitrogen, Carlsbad, CA, USA) according to the manufacturer’s instructions. cDNA was synthesized and messenger RNA (mRNA) expression levels of selected genes were quantified, as per our previous study^21^. Specific mouse primers for selected genes were designed (Table S1 in Supplementary Information). MxPro QPCR software was used for data analysis and the relative quantification of each mRNA was calculated using the 2^−ΔΔCt^ method with 18 S ribosomal RNA (18 S) as the endogenous control.

### Serum protein preparation and iTRAQ labelling

Four individual serum samples from the control and 5 mg PFOA/kg/d group, respectively, were randomly selected for iTRAQ analysis coupled with 2D LC-MS/MS analysis. High abundance serum proteins including albumin, IgG and IgA were removed using the ProteoMiner Protein Enrichment System (BioRad, PA, USA), and the protein concentration of each sample was then measured via the Bradford assay. One hundred micrograms of proteins from each sample were digested with trypsin (Promega, Madison, WI, USA) and labeled with iTRAQ reagents according to the kit protocols (ABI, Foster City, USA). The tryptic peptides were labeled with 8-plex iTRAQ reagents (isobaric tags 113, 114, 115 and 116 for the control, and 117, 118, 119 and 121 for the treated group, respectively).

### 2D LC-MS/MS analysis, data searching and quantification

For iTRAQ analysis, 2D LC-MS/MS methods were performed as per previous study^46^. The labeled peptides were pooled, and then dried by vacuum centrifugation. SCX chromatography was performed with a Shimadzu LC-20AB HPLC Pump system connected to a 4.6 × 250 mm Ultremex SCX column (Phenomenex, USA) to subsequently separate the mixed peptides. Mass spectroscopic (MS) analysis was performed using a Triple TOF 5600 mass spectrometer (AB Sciex, Concord, ON, Canada) coupled with a nanoACQuity HPLC system (Waters, USA). Microfluidic traps and nanofluidic columns packed with Symmetry C18 (5 μm, 180 μm × 20 mm) were utilized for online trapping and desalting, and nanofluidic columns packed with BEH130 C18 (1.7 μm, 100 μm × 100 mm) were employed in analytical separation. Data acquisition was performed with a TripleTOF 5600 System (AB Sciex, USA) fitted with a Nanospray III source (AB Sciex, USA).

The resulting MS/MS spectra were combined into one Mascot generic format (MGF) file and searched against the International Protein Index (IPI) mouse sequence databases (version 3.87, MOUSE, 59534 sequences) with MASCOT software (Matrix Science, London, UK; version 2.3.02). A protein with ≥ 1.5-fold difference and a *p*-value ≤ 0.05 was regarded as differentially expressed in the data. The methodological details are described in the Supplementary Information.

To better understand these differentially expressed proteins in relation to published literature, interactions among these proteins regarding biological pathways were determined using Pathway Studio software via the ResNet database (version 6.5, Ingenuity Systems, Inc.).

### Western blotting

Western blotting was performed to validate the iTRAQ results and detect levels of proteins related to glucose homeostasis from frozen tissues. Proteins were isolated from frozen liver, muscle or fat specimens using RIPA buffer (Thermo Scientific, USA) containing 1 mM phenylmethanesulfonyl fluoride (PMSF), with the supernatant then collected after centrifugation at 14000 × g for 15 min. Protein concentration was measured using a BCA protein assay kit (Tiangen, Beijing, China). The tissue protein samples (40 μg of each) or serum samples diluted to 1:20 with phosphate buffer solution (5 μL of each) were separated using 12% sodium dodecyl sulfate polyacrylamide gel electrophoresis (SDS-PAGE) and then transferred onto polyvinylidene fluoride (PVDF) membranes (Millipore, Billerica, MA, USA). Membranes were blocked with 5% bovine serum albumin (BSA) in Tris-buffered saline containing 0.1% Tween 20 (TBST) for 1 h and then incubated with the appropriate primary antibody diluted with 5% BSA in TBST containing 0.02% NaN_3_ at 4 °C overnight. Membranes were washed with TBST before incubation with horseradish peroxidase-coupled secondary antibodies (Santa Cruz Biotechnology, Santa Cruz, CA, USA) diluted with 5% non-fat milk or BSA in TBST at room temperature. Protein bands on membranes were detected using an enhanced chemiluminescence (ECL) system western blot detection kit (Tiangen, Beijing, China) and were visualized by exposure to X-ray film (Kodak, NY, USA). Membranes were subsequently washed and then incubated with β-tubulin for equal protein loading. Quantity One software (Bio-Rad, Hercules, CA, USA) was used to quantify the intensities of protein bands.

### Statistical analysis

Statistical analyses were performed using SPSS for Windows 17.0 software (SPSS, Inc., Chicago, IL, USA). Differences between only two treatment groups were evaluated by independent samples or paired *t-*test. Differences between treatments of more than two groups were evaluated using one-way analysis of variance (ANOVA) followed by Fisher's least significant difference (LSD) test. Data were represented as means with standard errors (mean ± S.E.). Results were considered statistically significant if the *p*-value was less than 0.05 (*) or 0.01(**).

## Additional Information

**How to cite this article**: Yan, S. *et al.* Perfluorooctanoic acid exposure for 28 days affects glucose homeostasis and induces insulin hypersensitivity in mice. *Sci. Rep.* **5**, 11029; doi: 10.1038/srep11029 (2015).

## Supplementary Material

Supplementary Information

## Figures and Tables

**Table 1:** Differentially expressed proteins in relation to diabetes or liver disease identified by iTRAQ^a^ in mouse serum after 5 mg/kg/d PFOA exposure for 28 d.

**Accession**	**Gene symbol**^b^	**Fold change (P/C)**^c^	**Accession**	**Gene symbol**^b^	**Fold change (P/C)**^c^
IPI00135189	Aacs	7.28	IPI00117042	Gfap	3.28
IPI00119114	Acadl	3.00	IPI00129243	Ggh	2.14
IPI00126248	Acly	2.75	IPI00319652	Gpx1	2.07
IPI00662244	Agl	2.39	IPI00469893	Habp2	0.64
IPI00626662	Aldh1a1	3.04	IPI00409148	Hp	5.34
IPI00124372	Aldh9a1	2.06	IPI00330804	Hsp90aa1	2.74
IPI00127206	Aldob	3.24	IPI00128522	Hspb1	3.67
IPI00127352	Ambp	1.62	IPI00312295	Ifnar2	1.52
IPI00128206	Angptl3	1.87	IPI00556721	Igfals	0.53
IPI00331221	Apoa5	0.55	IPI00313327	Igfbp2	1.86
IPI00119676	Apoc1	1.66	IPI00112485	Igfbp3	1.57
IPI00130382	Apom	0.50	IPI00113057	Klkb1	0.57
IPI00117914	Arg1	6.91	IPI00475209	Masp1	1.79
IPI00314788	Asl	1.74	IPI00111793	Nid1	2.08
IPI00130950	Bhmt	2.62	IPI00317356	Pon1	0.21
IPI00113996	Blvrb	1.80	IPI00338018	Postn	0.46
IPI00312058	Cat	4.37	IPI00121788	Prdx1	2.41
IPI00116283	Cct3	1.67	IPI00117910	Prdx2	2.68
IPI00135087	Cops5	1.74	IPI00131406	Psma7	1.91
IPI00111908	Cps1	2.12	IPI00319525	Pygl	1.67
IPI00138274	Cryab	4.18	IPI00225275	Pygm	3.75
IPI00119622	Csad	2.24	IPI00903401	Serpina1c	0.26
IPI00111013	Ctsd	1.75	IPI00118286	Sfn	2.56
IPI00108061	Cxcl12	0.61	IPI00125333	Slpi	0.66
IPI00331394	Dnpep	1.73	IPI00459493	Tcp1	1.52
IPI00307837	Eef1a1	1.53	IPI00127560	Ttr	0.48
IPI00466069	Eef2	1.97	IPI00118344	Ugdh	2.71
IPI00321617	Ephx2	4.82	IPI00131204	Ugp2	3.86
IPI00330843	F12	1.95	IPI00227299	Vim	2.02
IPI00307890	F7	0.52	IPI00129215	Vnn1	4.69
IPI00122312	Fgg	1.56	IPI00129240	Vtn	0.34

^a^iTRAQ was performed on four individual samples from each group, respectively.

^b^Full names of the proteins are referred to in Table S2.

^c^iTRAQ fold changes (P/C) were the average protein fold change of four individual samples. A protein with ≥ 1.5-fold or ≤ 0.66-fold difference and a *p*-value ≤ 0.05 was regarded as differentially expressed.
