# Supplementary material for: Perfluorooctanoic acid exposure for 28 days affects glucose homeostasis and induces insulin hypersensitivity in mice
Source: Sci Rep. 2015 Jun 12;5:11029. doi: 10.1038/srep11029 (PMC4464286; doi:10.1038/srep11029)
Supplement: Supplementary Information [file srep11029-s1.pdf]

Perfluorooctanoic acid exposure for 28 days affects glucose homeostasis in mice

*Shengmin Yan[#1], Hongxia Zhang[#1], Fei Zheng[2], Nan Sheng[1], Xuejiang Guo[3] and Jiayin Dai[1]**

a

b

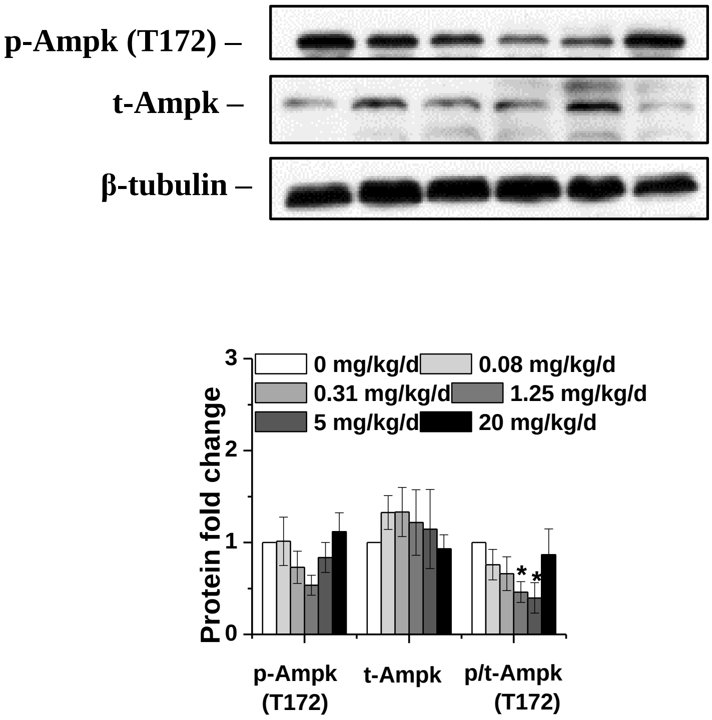

Fig. S1 Protein expression of phospho-AMPK (T172) and total AMPK in livers of mice after 28 d exposure to PFOA. (a) Representative western blots of phospho-AMPK and total AMPK. (b) Relative fold change of band densities (n = 3). Data are means ± SE. Significantly different from control group (*p < 0.05).

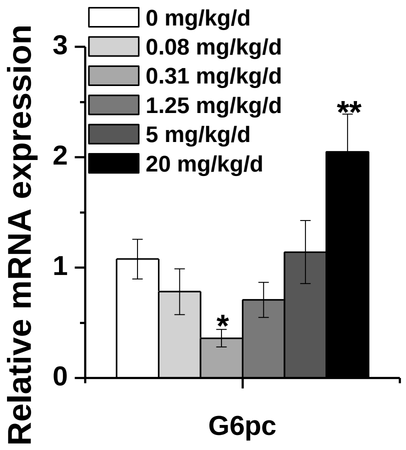

Fig. S2 Relative mRNA level of glucose-6-phosphatase catalytic subunit (G6PC) in livers of mice after 28 d exposure to PFOA (n = 6).

a

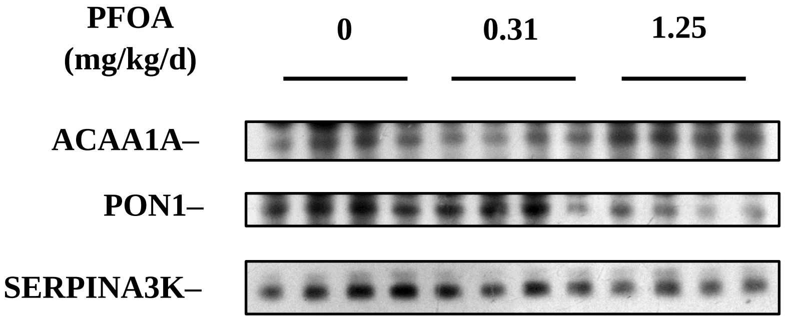

b

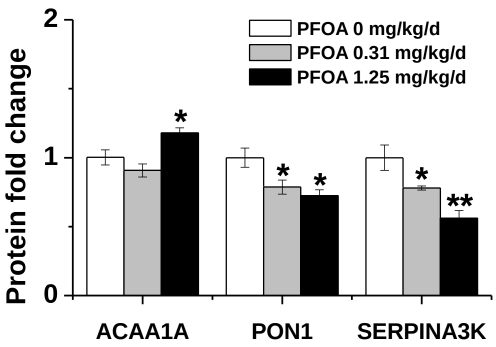

Fig. S3 Protein expression of ACAA1A, PON1 and SERPINA3K in serum of mice after 28 d exposure to PFOA at the dose of 0.31 and 1.25 mg/kg/d. (a) Representative western blots of ACAA1A, PON1 and SERPINA3K. (b) Relative fold change of band densities (*n* = 4). Data are means ± SE. Significantly different from control group (*p < 0.05, **p < 0.01).

Table S1 Primers for qPCR

**MOUSE**	**NM**	**(bp)**	**Forward (5' to 3')**	**Reverse (5' to 3')**
**18S**	NR_003278.3	159	GCCGTTCTTAGTTGGTGGA	GACCTGTTATTGCTCAATCTCG
**G6pc**	NM_008061.3	173	CGACTCGCTATCTCCAAGTGA	GTTGAACCAGTCTCCGACCA

Table S2 Alteration of body weight (BW), liver weight, white adipose tissue weight (WAT), brown adipose tissue weight (BAT) and serum biochemical levels (n = 10) after PFOA exposure, data are means ± SE, significantly different from control group (**p < 0.01).

	**PFOA (mg/kg/d)**	
	**0**	**5**
**BW before treatment (g)**	24.57 ± 0.42	24.43 ± 1.04
**Absolute liver weight (g)**	1.14 ± 0.03	2.57 ± 0.09**
**Relative liver weight (%)**	4.51 ± 0.08	10.97 ± 1.13**
**Absolute gonadal WAT weight (g)**	0.32 ± 0.02	0.06 ± 0.02**
**Relative gonadal WAT weight (%)**	1.28 ± 0.09	0.28 ± 0.07**
**Absolute inguinal WAT weight (g)**	0.25 ± 0.01	0.12 ± 0.02**
**Relative inguinal WAT weight (%)**	0.98 ± 0.05	0.54 ± 0.07**
**Absolute interscapular BAT weight (g)**	0.10 ± 0.01	0.04 ± 0.01**
**Relative interscapular BAT weight (%)**	0.41 ± 0.02	0.20 ± 0.01**
**ALT (IU/L)**	24.00±0.71	228.20±18.09**
**AST (IU/L)**	70.40±4.52	166.90±15.29**
**ALB (g/L)**	21.99±0.37	23.90±0.39**
**ALP (IU/L)**	59.50±1.69	338.60±20.86**
**TBA (μmol/L)**	1.17±0.11	6.47±1.04**
**T-CHO (mmol/L)**	3.16±0.07	2.79±0.10**
**TG (mmol/L)**	2.01±0.14	1.29±0.09**
**HDL (mmol/L)**	3.32±0.08	2.70±0.10**
**LDL (mmol/L)**	0.20±0.01	0.26±0.01**
**LDH (U/L)**	624.80±35.80	1082.40±36.64**

Table S3. Differential Expressed Proteins Identified by iTRAQ[a] in Mouse Serum after 5 mg/kg/d PFOA Exposure for 28 Days

**Accession**	**Gene symbol**	**Full name**	**Fold change (P/C)[b]**
IPI00754336	-	13 kDa protein	0.45
IPI00108830	-	Ig heavy chain V region HPCM6	0.63
IPI00661618	-	13 kDa protein	1.63
IPI00990967	-	3 kDa protein	2.33
IPI00127109	1700009N14Rik	Uncharacterized protein	1.53
IPI00135189	Aacs	Acetoacetyl-CoA synthetase	7.28
IPI00121833	Acaa1a	3-ketoacyl-CoA thiolase A, peroxisomal	3.27
IPI00122139	Acaa1b	3-ketoacyl-CoA thiolase B, peroxisomal	6.27
IPI00226430	Acaa2	3-ketoacyl-CoA thiolase, mitochondrial	3.78
IPI00119114	Acadl	Long-chain specific acyl-CoA dehydrogenase, mitochondrial	3.00
IPI00126248	Acly	ATP-citrate synthase isoform 1	2.75
IPI00221400	Adh1	Alcohol dehydrogenase 1	4.54
IPI00308217	Adsl	Adenylosuccinate lyase	1.99
IPI00662244	Agl	Uncharacterized protein	2.39
IPI00230440	Ahcy	Adenosylhomocysteinase	2.50
IPI00553798	Ahnak	AHNAK nucleoprotein isoform 1	1.69
IPI00128209	Ak1	Isoform 1 of Adenylate kinase isoenzyme 1	3.86
IPI00626662	Aldh1a1	Retinal dehydrogenase 1	3.04
IPI00230084	Aldh7a1	Isoform 1 of Alpha-aminoadipic semialdehyde dehydrogenase	2.68
IPI00124372	Aldh9a1	4-trimethylaminobutyraldehyde dehydrogenase	2.06
IPI00127206	Aldob	Fructose-bisphosphate aldolase B	3.24
IPI00127352	Ambp	Protein AMBP	1.62
IPI00128206	Angptl3	Angiopoietin-related protein 3	1.87
IPI00127754	Apbh	Androgen-binding protein eta precursor	0.62
IPI00309214	Apcs	Serum amyloid P-component	2.04
IPI00331221	Apoa5	Apolipoprotein A-V	0.55
IPI00119676	Apoc1	Apolipoprotein C-I	1.66
IPI00130382	Apom	Apolipoprotein M	0.50
IPI00117914	Arg1	Arginase-1	6.91
IPI00314788	Asl	Argininosuccinate lyase	1.74
IPI00130950	Bhmt	Betaine-homocysteine S-methyltransferase 1	2.62
IPI00113996	Blvrb	Flavin reductase	1.80
IPI00132388	Bzw1	Basic leucine zipper and W2 domain-containing protein 1	1.68
IPI00407502	C1ra	Complement C1r-A subcomponent	0.55
IPI00653675	C1s	Complement C1s-A subcomponent	0.56
IPI00134808	C4bp	C4b-binding protein	0.44
IPI00314270	C6	Complement component 6	0.57

IPI00230718	C9	Complement component C9	0.19
IPI00135186	Calu	Calumenin	0.59
IPI00896727	Cand1	Cullin-associated NEDD8-dissociated protein 1	1.55
IPI00312058	Cat	Catalase	4.37
IPI00116283	Cct3	T-complex protein 1 subunit gamma	1.67
IPI00331174	Cct7	T-complex protein 1 subunit eta	1.59
IPI00469268	Cct8	T-complex protein 1 subunit theta	1.55
IPI00266188	Cfl2	Cofilin-2	2.99
IPI00128682	Clec11a	C-type lectin domain family 11 member A	0.35
IPI00320239	Clec3b	Tetranectin	0.40
IPI00315879	Cndp2	Cytosolic non-specific dipeptidase	1.69
IPI00757292	Colec11	Isoform 2 of Collectin-11	1.87
IPI00135087	Cops5	COP9 signalosome complex subunit 5	1.74
IPI00271262	Cpamd8	Murinoglobulin-2	0.44
IPI00111908	Cps1	Carbamoyl-phosphate synthase [ammonia], mitochondrial	2.12
IPI00138274	Cryab	Alpha-crystallin B chain	4.18
IPI00119622	Csad	Cysteine sulfinic acid decarboxylase	2.24
IPI00111013	Ctsd	Cathepsin D	1.75
IPI00108061	Cxcl12	Isoform Alpha of Stromal cell-derived factor 1	0.61
IPI00230113	Cyb5	Cytochrome b5	1.99
IPI00310669	Dak	Bifunctional ATP-dependent dihydroxyacetone kinase/FAD-AMP lyase (cyclizing)	4.07
IPI00331394	Dnpep	Aspartyl aminopeptidase isoform a	1.73
IPI00138251	Dpt	Dermatopontin	0.50
IPI00307837	Eef1a1	Elongation factor 1-alpha 1	1.53
IPI00118875	Eef1d	Isoform 1 of Elongation factor 1-delta	1.91
IPI00466069	Eef2	Elongation factor 2	1.97
IPI00118018	Ehbp1l1	EH domain-binding protein 1-like protein 1 isoform a	1.94
IPI00187443	Eif5	Eukaryotic translation initiation factor 5	2.80
IPI00108125	Eif5a	Eukaryotic translation initiation factor 5A-1	1.97
IPI00125514	Entpd5	Ectonucleoside triphosphate diphosphohydrolase 5	2.30
IPI00321617	Ephx2	Isoform 1 of Epoxide hydrolase 2	4.82
IPI00330843	F12	Coagulation factor XII	1.95
IPI00307890	F7	Coagulation factor VII	0.52
IPI00111807	F8	Coagulation factor VIII	1.50
IPI00230432	Fbln1	Isoform C of Fibulin-1	0.54
IPI00122312	Fgg	Uncharacterized protein	1.56
IPI00117042	Gfap	Isoform 1 of Glial fibrillary acidic protein	3.28
IPI00129243	Ggh	Isoform I of Gamma-glutamyl hydrolase	2.14
IPI00758039	Gm4684	Uncharacterized protein	0.24
IPI00788443	Gm4788	Complement factor H-related protein C isoform 1	0.29
IPI00379634	Gm5325	Major allergen I polypeptide chain 1-like	0.21
IPI00606140	Gm5895	Uncharacterized protein	0.25
IPI00319652	Gpx1	Glutathione peroxidase 1	2.07

IPI00321666	H2-Q10	H-2 class I histocompatibility antigen, Q10 alpha chain	2.35
IPI00469893	Habp2	Isoform 2 of Hyaluronan-binding protein 2	0.64
IPI00316314	Hacl1	2-hydroxyacyl-CoA lyase 1	2.46
IPI00407339	Hist1h4m	Histone H4	2.05
IPI00224575	Hnrnpk	Isoform 2 of Heterogeneous nuclear ribonucleoprotein K	1.88
IPI00409148	Hp	Haptoglobin	5.34
IPI00331628	Hsd17b4	Peroxisomal multifunctional enzyme type 2	4.64
IPI00330804	Hsp90aa1	Heat shock protein HSP 90-alpha	2.74
IPI00554929	Hsp90ab1	Heat shock protein HSP 90-beta	2.14
IPI00128522	Hspb1	Isoform A of Heat shock protein beta-1	3.67
IPI00312295	Ifnar2	Isoform 3 of Interferon alpha/beta receptor 2	1.52
IPI00556721	Igfals	Insulin-like growth factor-binding protein complex acid labile subunit	0.53
IPI00313327	Igfbp2	Insulin-like growth factor-binding protein 2	1.86
IPI00112485	Igfbp3	Insulin-like growth factor-binding protein 3	1.57
IPI00137939	Igk-V19-17	Ig kappa chain V19-17	1.73
IPI00626994	Ipo5	Isoform 1 of Importin-5	1.61
IPI00227834	Itih2	Inter-alpha-trypsin inhibitor heavy chain H2	1.76
IPI00124725	Itih3	Inter-alpha-trypsin inhibitor heavy chain H3	2.72
IPI00119818	Itih4	Inter alpha-trypsin inhibitor, heavy chain 4 isoform 2	0.62
IPI00113057	Klkb1	Plasma kallikrein	0.57
IPI00403040	Ldb3	LIM domain-binding protein 3 isoform c	2.40
IPI00475209	Masp1	Isoform 2 of Mannan-binding lectin serine protease 1	1.79
IPI00128518	Mat1a	S-adenosylmethionine synthase isoform type-1	3.05
IPI00466733	Minpp1	Multiple inositol polyphosphate phosphatase 1	1.53
IPI00604945	Mpst	3-mercaptopyruvate sulfurtransferase	1.53
IPI00122862	Mthfd1	C-1-tetrahydrofolate synthase, cytoplasmic	1.90
IPI00742385	Mup3	Uncharacterized protein	0.24
IPI00750595	Myl9	Myosin regulatory light polypeptide 9	0.67
IPI00123199	Nap1l1	Nucleosome assembly protein 1-like 1	2.24
IPI00133977	Nap1l4	Putative uncharacterized protein	1.65
IPI00136134	Ndrg2	Isoform 1 of Protein NDRG2	2.11
IPI00127280	Ngp	Neutrophilic granule protein	2.08
IPI00111793	Nid1	Nidogen-1	2.08
IPI00453489	Oit3	Isoform 1 of Oncoprotein-induced transcript 3 protein	1.52
IPI00331541	Pfkm	6-phosphofructokinase, muscle type	2.81
IPI00310059	Pigr	Polymeric immunoglobulin receptor	1.68
IPI00127407	Plod1	Procollagen-lysine,2-oxoglutarate 5-dioxygenase 1	1.66
IPI00317356	Pon1	Serum paraoxonase/arylesterase 1	0.21
IPI00338018	Postn	Isoform 1 of Periostin	0.46
IPI00118736	Ppm1b	Protein phosphatase 1B isoform 1	1.97
IPI00121788	Prdx1	Peroxiredoxin-1	2.41
IPI00117910	Prdx2	Peroxiredoxin-2	2.68
IPI00652394	Prg4	Isoform D of Proteoglycan 4	1.76

IPI00133222	Proz	Vitamin K-dependent protein Z	0.60
IPI00283862	Psma1	Proteasome subunit alpha type-1	1.93
IPI00331644	Psma3	Proteasome subunit alpha type-3	1.64
IPI00277001	Psma4	Proteasome subunit alpha type-4	2.02
IPI00131407	Psma5	Proteasome subunit alpha type-5	2.10
IPI00131845	Psma6	Proteasome subunit alpha type-6	1.94
IPI00131406	Psma7	Proteasome subunit alpha type-7	1.91
IPI00113845	Psmb1	Proteasome subunit beta type-1	1.78
IPI00128945	Psmb2	Proteasome subunit beta type-2	1.98
IPI00314467	Psmb3	Proteasome subunit beta type-3	2.07
IPI00129512	Psmb4	Proteasome subunit beta type-4	1.89
IPI00317902	Psmb5	Proteasome subunit beta type-5	2.70
IPI00119239	Psmb6	Proteasome subunit beta type-6	2.43
IPI00136483	Psmb7	Proteasome subunit beta type-7	1.96
IPI00133206	Psmc3	26S protease regulatory subunit 6A	1.60
IPI00222515	Psmd11	26S proteasome non-ATPase regulatory subunit 11	1.66
IPI00114667	Psmd7	26S proteasome non-ATPase regulatory subunit 7	1.53
IPI00124223	Psme1	Proteasome activator complex subunit 1	1.58
IPI00131763	Psp	Parotid secretory protein	0.44
IPI00319525	Pygl	Glycogen phosphorylase, liver form	1.67
IPI00225275	Pygm	Glycogen phosphorylase, muscle form	3.75
IPI00461022	Qpct	Isoform 1 of Glutaminyl-peptide cyclotransferase	1.80
IPI00128556	Qprt	Nicotinate-nucleotide pyrophosphorylase [carboxylating]	2.85
IPI00137227	Rab2a	Ras-related protein Rab-2A	1.63
IPI00109324	Retnla	Resistin-like alpha	0.55
IPI00139795	Rplp2	60S acidic ribosomal protein P2	2.95
IPI00123604	Rpsa	40S ribosomal protein SA	1.70
IPI00123349	Sec23a	Protein transport protein Sec23A	2.06
IPI00903401	Serpina1c	Alpha-1-antitrypsin 1-3	0.26
IPI00131830	Serpina3k	Serine protease inhibitor A3K	0.23
IPI00135635	Serpina3m	Serine protease inhibitor A3M	0.49
IPI00118286	Sfn	14-3-3 protein sigma	2.56
IPI00125333	Slpi	Antileukoproteinase	0.66
IPI00226515	Tagln	Transgelin	1.82
IPI00125778	Tagln2	Transgelin-2	1.57
IPI00459493	Tcp1	Isoform 1 of T-complex protein 1 subunit alpha	1.52
IPI00127560	Ttr	Transthyretin	0.48
IPI00125135	Ube2d2	Ubiquitin-conjugating enzyme E2 D2	1.69
IPI00165854	Ube2n	Ubiquitin-conjugating enzyme E2 N	2.03
IPI00113257	Ube2v1	Isoform 1 of Ubiquitin-conjugating enzyme E2 variant 1	2.16
IPI00118344	Ugdh	UDP-glucose 6-dehydrogenase	2.71
IPI00131204	Ugp2	Isoform 1 of UTP--glucose-1-phosphate uridylyltransferase	3.86
IPI00113214	Usp5	Ubiquitin carboxyl-terminal hydrolase 5	1.77
IPI00126072	Vat1	Synaptic vesicle membrane protein VAT-1 homolog	1.51

IPI00227299	Vim	Vimentin	2.02
IPI00129215	Vnn1	Pantetheinase	4.69
IPI00129240	Vtn	Vitronectin	0.34
IPI00230707	Ywhag	14-3-3 protein gamma	2.20
IPI00408378	Ywhaq	Isoform 1 of 14-3-3 protein theta	1.60

[a] The iTRAQ was performed on four individual samples from each group respectively.

[b] The iTRAQ fold changes (P/C) were the average protein fold change of four individual samples. A protein with ≥1.5-fold or ≤0.67-fold difference and a p-value ≤0.05 was regarded as being differentially expressed.

Methods

2D LC- MS/MS analysis, database searching and quantification

The labeled peptides were pooled, and dried by vacuum centrifugation. SCX chromatography was performed with a Shimadzu LC-20AB HPLC Pump system connected to a 4.6 × 250 mm Ultremex SCX column (Phenomenex USA). The peptide mixtures were reconstituted with 4 mL of buffer A (25 mM NaH2PO4 in 25% CAN, pH 2.7), and then eluted at a flow rate of 1 mL/min with a gradient of buffer A for 10 min, 5–35% buffer B (25 mM NaH2PO4, 1 M KCl in 25% ACN, pH 2.7) for 11 min, and 35–80% buffer B for 1 min. Elution was monitored by measuring the absorbance at 214 nm, and fractions were collected every 1 min. The eluted peptides were pooled into 12 fractions, desalted with a Strata X C18 column (Phenomenex) and vacuum-dried.

Mass spectroscopic (MS) analysis was performed using a Triple TOF 5600 mass spectrometer (AB SCIEX, Concord, ON) coupled with a nanoACQuity HPLC system (Waters, USA). Microfluidic traps and nanofluidic columns packed with Symmetry C18 (5 μm, 180 μm × 20 mm) were utilized for online trapping and desalting, and nanofluidic columns packed with BEH130 C18 (1.7 μm, 100 μm × 100 mm) were employed in analytical separation. The mobile phases purchased from Thermo Fisher Scientific (USA) were composed of water/acetonitrile/formic acid (A: 98/2/0.1%; B: 2/98/0.1%). A portion of a 2.25 μg (9 μL) sample was loaded, trapped and desalted at a flow rate of 2 μL/min for 15 min with buffer A. Peptides were then separated using the following gradient at a flow rate of 300 nL/min: 5% B for 1 min, 5% to 35% B for 40 min, 35% to 80% B for 5 min, and 80% B for 5 min. Initial chromatographic conditions were restored after 2 min.

Data acquisition was performed with a TripleTOF 5600 System (AB SCIEX, USA) fitted with a Nanospray III source (AB SCIEX, USA). Data were acquired using an ion spray voltage of 2.5 kV. Survey scans were acquired in 250 ms and up to 30 product ion scans were collected if they exceeded a threshold of 120 counts per second (counts/s) with a 2+ to 5+ charge-state. A sweeping collision energy setting of 35 ± 5 eV coupled with iTRAQ adjusted rolling collision energy was applied to all

precursor ions for collision-induced dissociation. Dynamic exclusion was set for half of the peak width (18 s), and the precursor was then refreshed off the exclusion list.

The resulting MS/MS spectra were combined into one Mascot generic format (MGF) file and searched against the International Protein Index (IPI) mouse sequence databases (version 3.87, MOUSE, 59534 sequences) with MASCOT software (Matrix Science, London, U.K.; version 2.3.02). Only unique peptides used for protein quantification were chosen to quantify proteins. The search parameters were as follows: trypsin as the enzyme, with one missed cleavage allowed; a fixed modification of carbamidomethylation at Cys; variable modifications of oxidation at Met and iTRAQ 8-plex at Tyr; mass tolerance of 0.05 Da for peptide and 0.1 Da for fragment ions. An automatic decoy database search strategy was employed to estimate the false discovery rate (FDR). In the final search results, the FDR was less than 1.5%. iTRAQ 8-plex was chosen for quantification during the search. For protein identification, the filters were set as follows: significance threshold $p < 0.05$ (with 95% confidence) and ion score or expected cutoff less than 0.05 (with 95% confidence). For protein quantification, the filters were set as follows: “median” was chosen for protein ratio type; minimum precursor charge was set to 1 and minimum peptides were set to 2; only unique peptides were used to quantify proteins. Summed intensities were set as normalization, and outliers were removed automatically. The peptide threshold was set as above for identity threshold.

Abbreviations

PFAAs, perfluoroalkyl acids

PFOA, perfluorooctanoic acid

ALT, alanine aminotransferase

AST, aspartate aminotransferase

ALP, alkaline phosphatase

TCHO, total cholesterol

TG, triglyceride

PPAR, peroxisome proliferator activated receptor

G6PC, glucose-6-phosphatase, catalytic

PTEN, phosphatase and tensin homolog

18S, 18S ribosomal RNA

ACAA1A, 3-ketoacyl-CoA thiolase A

PON1, serum paraoxonase/arylesterase 1

SERPINA3K , serine protease inhibitor A3K

AMPK, 5'-AMP-activated protein kinase

AKT, serine/threonine-protein kinase

IRβ, insulin receptor β

PI3K, phosphatidylinositol 3- kinase

p110, phosphatidylinositol 4,5-bisphosphate 3-kinase catalytic subunit alpha isoform

p85, phosphatidylinositol 3-kinase regulatory subunit

PDK, 3-phosphoinositide-dependent protein kinase

GSK3β, glycogen synthase kinase 3-beta

4E-BP1, eukaryotic translation initiation factor 4E-binding protein 1

IGF, insulin like growth factor

IGFBP, insulin like growth factor binding proteins
